# Effects of Uncertainty of Outlet Boundary Conditions in a Patient-Specific Case of Aortic Coarctation

**DOI:** 10.1007/s10439-021-02841-9

**Published:** 2021-08-24

**Authors:** Maria Nicole Antonuccio, Alessandro Mariotti, Benigno Marco Fanni, Katia Capellini, Claudio Capelli, Emilie Sauvage, Simona Celi

**Affiliations:** 1 BioCardioLab, Bioengineering Unit - Heart Hospital, Fondazione Toscana “G. Monasterio”, Massa, Italy; 2 Civil and Industrial Engineering Department, University of Pisa, Pisa, Italy; 3 Information Engineering Department, University of Pisa, Pisa, Italy; 4 Institute of Cardiovascular Science, University College of London, London, UK

**Keywords:** Aortic coarctation, Computational fluid dynamics, Windkessel model, Uncertainty quantification, Magnetic resonance imaging

## Abstract

Computational Fluid Dynamics (CFD) simulations of blood flow are widely used to compute a variety of hemodynamic indicators such as velocity, time-varying wall shear stress, pressure drop, and energy losses. One of the major advances of this approach is that it is non-invasive. The accuracy of the cardiovascular simulations depends directly on the level of certainty on input parameters due to the modelling assumptions or computational settings. Physiologically suitable boundary conditions at the inlet and outlet of the computational domain are needed to perform a patient-specific CFD analysis. These conditions are often affected by uncertainties, whose impact can be quantified through a stochastic approach. A methodology based on a full propagation of the uncertainty from clinical data to model results is proposed here. It was possible to estimate the confidence associated with model predictions, differently than by deterministic simulations. We evaluated the effect of using three-element Windkessel models as the outflow boundary conditions of a patient-specific aortic coarctation model. A parameter was introduced to calibrate the resistances of the Windkessel model at the outlets. The generalized Polynomial Chaos method was adopted to perform the stochastic analysis, starting from a few deterministic simulations. Our results show that the uncertainty of the input parameter gave a remarkable variability on the volume flow rate waveform at the systolic peak simulating the conditions before the treatment. The same uncertain parameter had a slighter effect on other quantities of interest, such as the pressure gradient. Furthermore, the results highlight that the fine-tuning of Windkessel resistances is not necessary to simulate the *post*-stenting scenario.

## Introduction

The Aortic Coarctation (CoA) is a Congenital Heart Defect (CHD) which occurs in four newborns out of 10,000 and accounts for approximately 5–10% of all CHDs.^8^ This is an alteration in the shape of the aorta that appears narrowed typically in the thoracic district, distally to the origin of the left subclavian artery, near the ductal structure. The narrowing affects both the shape and functionality of the aorta. Indeed, in CoA patients, the physiological blood flow is altered, resulting in high blood pressure in the upper part of the body.^15^ The most common hemodynamic analysis involves measuring the pressure gradient ($\Delta$*P*) across the coarctation site *via* invasive catheterization. The guideline for CoA treatment recommends procedural treatment when $\Delta{P}\geqslant {\text{20 mmHg}}$ at rest.^4^ Despite being considered a clinical gold standard, catheterization is an invasive procedure and may result in aortic dissection and death.^15^ To reduce these potential adverse events, the European Society of Cardiology guidelines recommend non-invasive assessments of CoA severity using imaging techniques.^4^ Over the last decade, personalised Computational Fluid Dynamics (CFD) models have been investigated as a tool to improve the understanding and clinical outcome of cardiovascular disorders. In this context, the pressure drop across the CoA district can be derived from the numerical solution of the Navier-Stokes equations with the use of Magnetic Resonance Imaging (MRI) velocity field data.^3^,^11^,^17^,^23^ Advances in image acquisition and numerical simulations have resulted in increasingly realistic patient-specific models. To make a digital twin of the patient, we need the geometric accuracy, and input and output boundary conditions. Among these, the output boundary conditions remain the most challenging to obtain from *in-vivo* data and to replicate in a computational environment.^20^ Simplified *static* outlet boundary conditions (BCs), such as constant pressure or prescribed flow split, have been replaced by more physiologically-accurate *dynamic* three-element Windkessel models (3WKMs).^6^,^7^,^22^ The 3WKM consists of a proximal resistance, ${R_{\text{p}}}$, in series with a parallel arrangement of capacitance, *C*, and distal resistance, ${R_{\text{d}}}$. The lumped parameters system is then coupled to the 3D domain to solve the CFD simulations.^17^ In the case of aortic coarctation, the flow patterns are more complex because the presence of the narrowing offers additional resistance to the blood flow. Therefore, it is necessary to tune the 3WKMs to fit the clinical data. Several strategies have been developed to adequately simulate hemodynamic conditions in the presence of aortic coarctations. Kim *et al.* introduced a method to strongly couple a lumped parameter heart model to a 3D finite element model of the aorta with CoA.^16^ Itu *et al.* proposed a CFD-based approach for non-invasive hemodynamic assessment of pre- and post-operative coarctation based on 3WKM.^14^ Pant *et al.* employed the unscented Kalman filter (UKF), which is a sequential estimation method, to evaluate the Windkessel parameters at the outlets of a CoA.^21^ More recently, Marx *et al.* developed a methodology to identify the parameters of a three-element Windkessel model of the left ventricle afterload due to the presence of CoA. A strong dependence of 3WKMs on input uncertainty was revealed.^19^ In the cardiovascular field, there are multiple sources of uncertainty to consider and that propagate through the model.^10^ In this scenario, computational simulations can determine how robust the simulation results are to the variation of input parameters.

This study aims to quantify the effects of outlet boundary conditions in modelling the hemodynamics of aortic coarctation and to propose a novel methodology to tune the 3WKMs, given patient-specific measurements of flow rate and pressure. We used generalised polynomial chaos expansion to perform the uncertainty quantification and expressed stochastic results as stochastic standard deviations, and probability distribution functions. Thus, because the hemodynamics of a CoA model is highly dependent on the peculiar geometry, the effect of geometry before and after the stenting procedure was considered in the present work.

## Theoretical Background of gPC

We performed a stochastic sensitivity analysis using the generalized Polynomial Chaos (gPC) method. This strategy allows obtaining a continuous response surface in the parameter space, using a few deterministic simulations. The basic principle of the gPC approach is the projection of a given stochastic response in terms of an orthogonal polynomial basis.^27^

Adopting term-base indexing, the gPC expansion for a given quantity of interest, *X*, can be expressed as:

1 $$\begin{aligned} X(\omega ) = \displaystyle {\sum _{k=0}^{\infty }} a_k \Phi _k(\varvec{\xi }(\omega )) \end{aligned}$$

where $X(\omega )$ is the random process, $\varvec{\xi }(\omega )$ is the vector consisting of the independent random variables (i.e. the set of considered uncertain parameters), $\Phi _k(\varvec{\xi })$ is the gPC polynomial of index *k* and $a_k$ is the corresponding Galerkin projection coefficient. The response surface is obtained by a truncation of the above expansion (Eq. 1) to a finite limit $\Lambda$. Applying full tensor-product polynomial expansion, $\Lambda$ is computed as follows:

2 $$\begin{aligned} \Lambda = \prod _{i=1}^M(P_i+1)-1 \end{aligned}$$

*M* is the number of the uncertain parameters, and $P_i$ is the maximum polynomial order chosen for the *i*-th parameter. Thanks to the orthogonality of the polynomial basis, the coefficients $a_k$ are obtained as:

3 $$\begin{aligned} a_k= \frac{\langle X, \Phi _k \rangle }{\langle \Phi _k, \Phi _k \rangle } = \frac{1}{\langle \Phi _k, \Phi _k \rangle } \int _{\text{supp}}{{\varvec{\xi }}} X \Phi _k \rho (\varvec{\xi }) {\textit{d}} \varvec{\xi } \end{aligned}$$

$\rho (\varvec{\xi })$ is the weight function associated with the selected polynomial family. In the present work, the above integrals were computed through the Gaussian quadrature. The polynomial family, $\Phi _k$, must be *a priori* specified and its choice affects the speed of the convergence of the gPC expansion. When dealing with Gaussian quadrature, an optimal polynomial family has a weight function analogous to the probability measure of the random variables. The optimal polynomial family thus depends on the PDF distribution chosen for the uncertain parameters.

## Materials and Methods

This section illustrates:

1. Patient’s data acquisition;
2. Image processing for 3D model creation and flow extraction;
3. Computational setup and definition of outlet boundary conditions (OBCs);
4. Definition of the stochastic analysis for the OBCs.

### Clinical Data

The study is based on a clinical dataset of a 12-years-old patient affected by aortic coarctation with a minimum coarctation diameter equal to 0.98 cm. The dataset was acquired at the Great Ormond Street Hospital (London, UK) as part of the clinical routine procedure. The use of retrospectively collected image data for research purposes was approved by the Institute of Child Health/Great Ormond Street Hospital Research Ethics Committee, and written consent was obtained from all subjects or parents/legal guardians (Ref: 06/Q0508/124).

**Figure 1:**
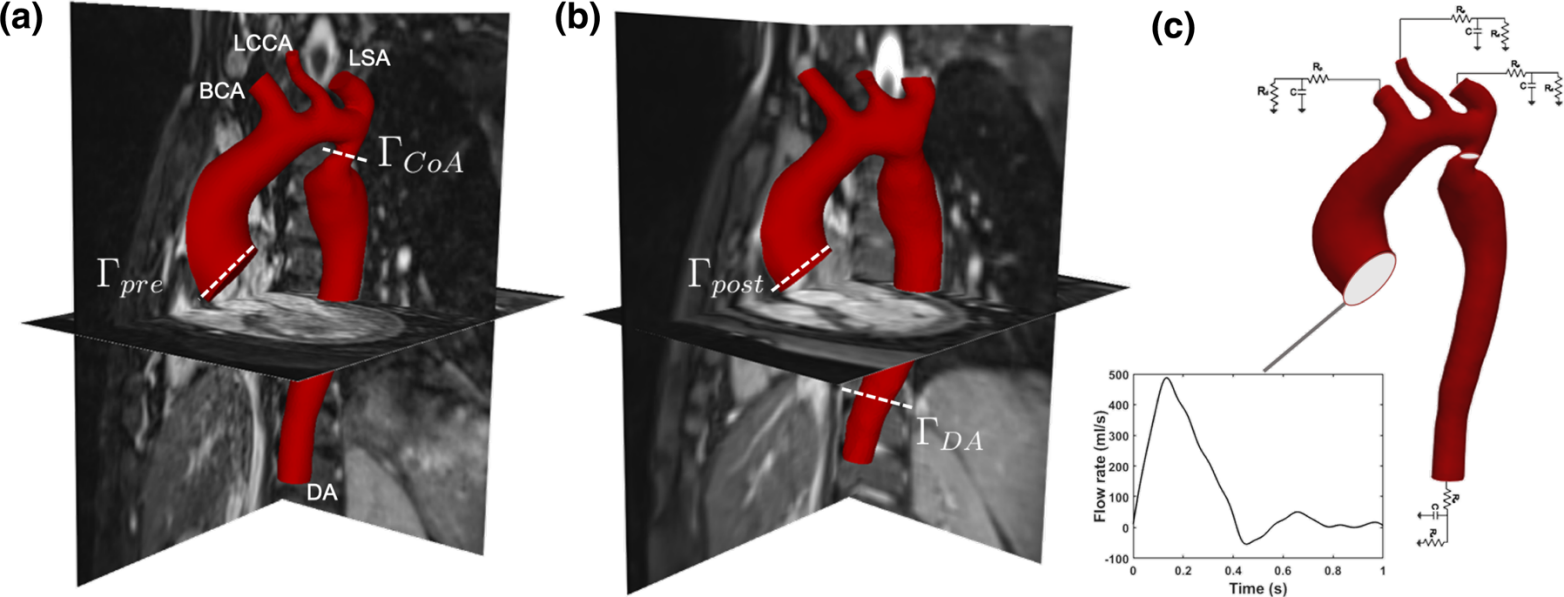
Segmented 3D geometry before (${M_{\text{pre}}}$) (**a**), and after (${M_{\text{post}}}$) (**b**) the stenting procedure with the identification of the planes used for the PC-MRI acquisitions. Time-dependent parabolic flow rate waveform (**c**) prescribed as inlet boundary condition at plane $\Gamma _{\text{pre}}$ before, and at plane $\Gamma _{\text{post}}$ after the stenting procedure. The 3WKMs are coupled at the four outlets: Brachiocephalic Artery (BCA), Left Common Carotid Artery (LCCA), Left Subclavian Artery (LSA) and Descending Aorta (DA).

The dataset included MRI acquisitions and catheter pressure measurements before and after the endovascular stenting procedure (hereafter referred to as *pre-SP* and *post-SP*). 2D Phase-Contrast MRI (PC-MRI) data and 3D -whole-heart- sequences were acquired using a Siemens Avanto 1.5 T (Siemens AG, Germany) MRI scanner. The velocity encoding was set at 200 cm/s for all directions, slice thickness was 5 mm, temporal resolution was equal to 32 ms, and echo time to 2.08 ms. PC images were acquired *pre-SP* and *post-SP* in four different transverse planes. Aortic planes ($\Gamma _{\text{pre}}$ and $\Gamma _{\text{post}}$, respectively) were acquired during both acquisitions to measure the inflow blood profile. To define the outflow conditions, the coarctation plane ($\Gamma _{\text{CoA}}$) was used before the stenting procedure, and a plane at the diaphragm level of the descending aorta ($\Gamma _{\text{DA}}$) was used after the stenting procedure. Figures 1a–1b illustrate the four acquisition planes. The cardiac cycle duration was 0.7 s before the endovascular stenting procedure, and it was equal to 1 s after the procedure.

In Table 1, the pressure values recorded through the cardiac catheterization *pre-SP* and *post-SP* are reported. These values are measured at two different sections $\Gamma _{\text{pre}}$/$\Gamma _{\text{CoA}}$, and $\Gamma _{\text{post}}$/$\Gamma _{\text{CoA}}$, respectively.

**Table 1:** Pressure (mmHg) acquired with respect to $\Gamma _{\text{pre}}$/$\Gamma _{\text{CoA}}$ and $\Gamma _{post}$/$\Gamma _{CoA}$.

	$P_{\text{max}}$	$P_{\text{min}}$	$P_{\text{meam}}$
$\Gamma _{\text{pre}}$/$\Gamma _{\text{CoA}}$	97/75	64/67	80/71
$\Gamma _{\text{post}}$/$\Gamma _{\text{CoA}}$	85/75	63/62	74/68

### Image Processing

The segmentation of the 3D -whole-heart- images was performed to extract the two geometries before, i.e. ${M_{\text{pre}}}$ (Fig. 1a) and after, i.e. ${M_{\text{post}}}$ (Fig. 1b) the stenting procedure. The open-source software ITK-SNAP was used to create the 3D models.^30^ The coarctation cross-sectional area was equal to 0.75 cm$^2$, as measured from ${M_{\text{pre}}}$, whereas the aortic inlet and outlet cross-sections were 9.2 cm$^2$ and 2.27 cm$^2$, respectively. Pre-operative cross-sectional areas were 1.44 cm$^2$, 0.28 cm$^2$ and 1.36 cm$^2$ for the Brachiocephalic Artery (*BCA*), Left Common Carotid Artery (*LCCA*), and Left Subclavian Artery (*LSA*), respectively. After the procedure, cross-sectional areas were 1.06 cm$^2$, 0.28 cm$^2$ and 1.36 cm$^2$ for *BCA*, *LCCA* and *LSA*, respectively. The flow rate waveforms were extracted at the four acquisition planes (Figs. 1a–1b) from the PC-MRI sequences using the freely available software Segment (Medviso AB, Lund).^12^ The flow extraction was based on an automatic selection of a flow’s region of interest (ROI), with further automatic vessel tracking and manual refinement for each phase of the MR acquisitions. Three different operators, with more than five years of experience, performed the manual refinement of the flow and the mean flow rate waveform was used. Partial volume effects near the wall were compensated performing a specific refinement of the ROI. In addition, minor aliasing effects associated to the setting were filtered out. The flow information extracted at $\Gamma _{\text{pre}}$ is reported in Fig. 1c. The waveforms extracted at $\Gamma _{\text{pre}}$ and $\Gamma _{\text{post}}$ of Figs. 1a–1b were prescribed as the inlet boundary conditions of the CFD simulations, whereas the waveforms at $\Gamma _{\text{CoA}}$ and $\Gamma _{\text{DA}}$ were used to validate the results of the numerical simulations. A time-varying flow rate waveform, assuming a parabolic velocity profile, was set at the inlet.

### Governing Equations and Computational Set-Up

The numerical simulations were performed using the open-source software SimVascular (Stanford University, California).^18^ In agreement with most studies in the literature, we considered the blood flow as an incompressible fluid with Newtonian rheology, according to Eq. 4.^13^,^23^,^28^

4 $$\begin{aligned} \begin{matrix} \rho \big (\mathbf {u} \cdot \nabla \big ) \mathbf {u} + \nabla p-\mu \Delta \mathbf {u} =0 \\ \nabla \cdot \mathbf {u} = 0 \end{matrix} \end{aligned}$$

where $\rho$ is blood density, $\mathbf {u}$ is the fluid velocity vector, *p* is the blood pressure, and $\mu$ is the constant viscosity. The blood-mimicking fluid was modelled with viscosity $\mu$ = 4 cP, and density $\rho$ = 1060 kg/m$^3$. The discretization of the Navier-Stokes equations by the finite-element method, which involves the introduction of a numerical viscosity, could be considered as an implicit large-eddy simulation. In order to verify the possible presence of resolved turbulent fluctuations in the coarctation region, characterized by significant curvature of streamlines and flow acceleration, we analyzed the time signals of the flow variables in this locations. We did not notice significant deviations from a periodic behavior for all the considered signals meaning that no significant turbulence fluctuations were found corresponding to turbulence scales resolvable on the considered grids.

In both ${M_{\text{pre}}}$ and ${M_{\text{post}}}$ geometries, we classified the different portions of the boundary as follows: the physical wall of the aortic artery (i); the inflow sections $\Gamma _{\text{pre}}$ and $\Gamma _{\text{post}}$ (ii); and the set of (unconnected) outflow sections (iii). In our models, outlets include BCA, LCCA, LSA, and Descending Aorta (DA).

A no-slip condition was imposed between the fluid and the walls, which were assumed rigid for all simulations. The computational domain was discretized by using tetrahedral elements, a prism-layer at the wall boundary, consisting in three layers of prisms with a triangular base, and a specific refinement in the CoA region. After a mesh sensitivity analysis, based on WSS computation, the element size was set to 1.2 mm for both models. The grids consisted of 1.7 $\times$ $10^6$ tetrahedral elements for ${M_{\text{pre}}}$, and 1.6 $\times$ $10^6$ tetrahedral elements for $M_{\text{post}}$.

The physical time step and maximum convergence residuals were set to 0.002 s and $10^{-4}$, respectively, for both cases. The cardiac cycle period was set equal to 0.7 s to simulate the *pre-SP* conditions, and equal to 1 s to simulate the *post-SP* ones, according to the clinical data. To ensure temporal independence of the results, the isolated models were simulated for six cardiac cycles, and the results from the last cycle were used to reduce potential errors due to model initialization.

Since output flow data were not available, and blood flow is a function of the downstream vasculature, we adopted a 3WKM that includes two resistances and one compliance.^17^,^22^,^25^ In this work, the outlet boundary conditions were obtained by coupling the 3D model outputs with a lumped-parameter model, as shown in Fig. 1c. A critical step when using a surrogate model, such as 3WKM, is the identification of the lumped parameters $R_{\text{p}}$, $R_{\text{d}}$ and *C*. The approach we propose here to compute 3WKMs is based on an optimization and fine-tuning procedure, as explained below. To estimate the value of the proximal $R_{\text{p}}$ and distal $R_{\text{d}}$ resistances, as well as the capacitance *C* for each outlet, a specific workflow was developed based on two main steps:

- *3WKM-total*: an optimization problem was defined to calculate the total values of the resistances ($\overline{R}{_p}$, $\overline{R}{_d}$), and the compliance ($\overline{C}$);
- *3WKM-tuning*: the previously obtained values of resistances and capacitance were distributed to the outlets, according to their relative cross-sectional areas. The area of the CoA was adopted for the computation of the 3WKM at the descending aorta. An additional parameter ($\alpha$) was introduced to redistribute the blood flows at the outlets of the computational domain.

***3WKM-total -*** The first step was to calculate the three-element Windkessel equation in which the patient pressure was calculated using the patient flow rate waveform.^17^ The solution of the Windkessel equation is reported in Eq. 5, and establishes a relation between the pressure P(t) and the flow rate Q(t) that can be assumed to be valid in downstream sections:

5 $$\begin{aligned} P(t)= [P(0) - \overline{R}_{p}Q(0)]e^{-t/\tau } + \overline{R}_{p}Q(t) + \int _0^t \frac{e^{-(t-{{t^*}})/\tau }}{\overline{C}}Q(t^*) {\text{d}}t^* \end{aligned}$$

*P(t)* and *Q(t)* stand for the pressure waveforms calculated as a function of time and known flow rate, respectively, while the $\overline{R}{_p}$, $\overline{R}{_d}$, and $\overline{C}$ are the *overall* lumped parameters of the Windkessel model. According to the electrical analogy of Eq. 5, $\tau$ = $\overline{R}$_d_$\overline{C}$ is the time-constant, and it describes the velocity response of the system to variations in the input function. P(0) and Q(0) are the initial pressure and flow conditions. The computation of the lumped parameters to obtain an accurate pressure waveform is not a trivial task. The optimization algorithm is the MOGA-II Multi-Objective Genetic Algorithm, used here as a single-objective function. The space for each parameter ($R_p$, $R_d$, C) is discretized into intervals of uniform size. An initial population is generated through a pseudo-random Sobol sequence, using subsets of the specified discrete parameter values. The population evolves through the following reproduction operators: directional crossover, mutation and selection. The probability of directional crossover, mutation and selection are set to 0.5, 0.1 and 0.05. The Eq. 6 is chosen as the objective function to minimize. The patient-specific pressure and flow rate values, introduced in Sect. 3.2, are the input data of the optimization algorithm. In this framework, the following optimization problem is defined:

6 $$\begin{aligned} {\left\{ \begin{array}{ll} \min (\frac{1}{T} \int _0^T P(t){\text{d}}t-P_{\text{mean}})^2\\ |\max (P)-P_{\text{max}}|\le \varepsilon _{\text{max}}\\ |\min (P)-P_{\text{min}}|\le \varepsilon _{\text{min}}\\ l_{b}\le x \le u_b \end{array}\right. } \end{aligned}$$

$P_{\text{max}}$, $P_{\text{min}}$ and $P_{\text{mean}}$ are the maximum, minimum and mean patient’s pressure values at the ascending aorta given in Table 1, whereas *x* is the set of the optimization variables, namely $\overline{R}{_p}$, $\overline{R}{_d}$, and $\overline{C}$ returned by the algorithm and bounded within lower ($l_b$) and upper ($u_b$) values. Two tolerances ($\varepsilon _{\text{max}}$ = $\varepsilon _{\text{min}} = 0.001$) were chosen to obtain pressure values consistent with those of the patient.

***3WKM-tuning -*** Starting from previous studies, the values of the 3WKM at each outlet of the model were calculated according to the Eq. 7.^6^,^26^ This approach is based on the distribution of $\overline{R}{_p}$, $\overline{R}{_d}$, and $\overline{C}$ at the various outlets because $\overline{R}{_p}$, $\overline{R}{_d}$, and $\overline{C}$ are independent of the particular geometry. To calculate the 3WKM at the descending aorta, considering the additional resistance introduced by the coarctation, we switched from the standard approach given in Eq. 7 to a new Eq. 8.

7 $$\begin{aligned} {\left\{ \begin{array}{ll} R_{p{_i}} = \overline{R}_p \frac{A_{\text{tot}}}{A_i} \\ R_{d{_i}} = \overline{R}_d \frac{A_{\text{tot}}}{A_i} \\ C_{_i} = \overline{C} \frac{A_i}{A_{\text{tot}}} \\ \end{array}\right. } \end{aligned}$$

8 $$\begin{aligned} {\left\{ \begin{array}{ll} R_{p_{DA}} = \overline{R}_p \frac{A_{\text{tot}}}{A_{\text{CoA}}} \\ R_{d_{DA}} = \overline{R}_d \frac{A_{\text{tot}}}{A_{\text{CoA}}} \\ C_{DA} = \overline{C} \frac{A_{\text{CoA}}}{A_{\text{tot}}} \\ \end{array}\right. } \end{aligned}$$

In Eq. 7, $A_i$ is the area of the considered outlet ($A_i =A_{\text{BCA}},\; A_{\text{LCCA}},\; A_{\text{LSA}}, \; A_{\text{DA}}$), and $A_{\text{tot}}$ is the sum of all the areas of the outlets.^7^, ^29^$\overline{R}{_p}$, $\overline{R}{_d}$, and $\overline{C}$ are the Windkessel parameters obtained as described previously. In Eq. 8, the minimum cross-sectional area at the site of coarctation, $A_{\text{CoA}}$, is introduced. $A_{\text{CoA}}$ is then used to calculate the 3WKM at the descending aorta instead of the area of the descending aorta ($A_{\text{DA}}$). In this way, we considered the additional resistance to flow from a geometrical point of view. Since the total resistance of the system ($R_{\text{tot}}$ = $\overline{R}_p$ + $\overline{R}_d$) is constant, the Eq. 9 can be defined:

9 $$\begin{aligned} {\left\{ \begin{array}{ll} \frac{1}{R_{\text{tot}}} = \frac{1}{R_{DA_{\text{tot}}}} + \frac{1}{R_{BCA_{\text{tot}}}} + \frac{1}{R_{LCCA_{\text{tot}}}} + \frac{1}{R_{LSA_{\text{tot}}}} \\ {R_{p_{i}} = \overline{R}_{p}\frac{A_{\text{tot}}}{A_i}}\\ {R_{d_{i}} = \overline{R}_{d}\frac{A_{\text{tot}}}{A_i}}\\ \end{array}\right. } \end{aligned}$$

where *i* is the $i-th$ outlet (*BCA*, *LCCA*, *LSA* and *DA*).

To further tune the 3WKMs, a non-dimensional split value $\alpha$ can be introduced. Considering $\alpha$, the $R_p$ and $R_d$ at the supra-aortic branches can be written as follows (Eq. 10):

10 $$\begin{aligned} R_i =(1+\alpha ) R_{\text{tot}}\frac{A_{\text{tot}}}{A_i} \end{aligned}$$

where *i* indicates the *i-th* supra-aortic branch (*BCA*, *LCCA*, and *LSA*); $R_i=R_{p_{i}}+R_{d_{i}}$ at each supra-aortic branch; $A_i =(A_{\text{BCA}},\; A_{\text{LCCA}},\; A_{\text{LSA}})$, and $A_{\text{tot}}$ = $A_{\text{BCA}}$ + $A_{\text{LCCA}}$ + $A_{\text{LSA}}$ + $A_{\text{CoA}}$.

When the CoA resistance increases, the flow at the supra-aortic branches increases, and consequently, the flow at the descending aorta decreases. By substituting Eq. 10 in Eq. 9, the following Eq. 11 is obtained for $R_{\text{DA}}=R_{p_{\text{DA}}}+R_{d_{\text{DA}}}$ at the descending aorta:

11 $$\begin{aligned} R_{\text{DA}} = \frac{R_{\text{tot}}A_{\text{tot}}}{A_{\text{tot}}-\sum \limits _{i} \frac{A_{i}}{1+\alpha }} \end{aligned}$$

To keep a more compact notation, we assume:

12 $$\begin{aligned} k=\frac{A_{\text{CoA}}}{A_{\text{tot}}-\sum \limits _{i} \frac{A_{i}}{1+\alpha }} \end{aligned}$$

Hence, similarly to Eq. 10, it is possible to express the resistance of the descending aorta as it follows:

13 $$\begin{aligned} R_{\text{DA}} = k R_{\text{tot}}\frac{A_{\text{tot}}}{A_{\text{CoA}}} \end{aligned}$$

### gPC Analysis

The approach proposed here allowed us to evaluate the sensitivity of the output quantities of interest to the parameter $\alpha$, used to tune the 3WKMs (see Eq. 10). For this purpose, we assumed a uniform Probability Density Function (PDF) since it was not possible to define an *a-priori* hypothesis. Consequently, Legendre polynomials were used. As mentioned in Section 3.4, the range of the parameter $\alpha$ was chosen based on preliminary simulations to guarantee that a fairly wide range of variation of the flow waveform across the coarctation was explored. Deterministic simulations were performed for ${\alpha }=-0.15, -0.13, -0.10, -0.08$. The convergence of the truncated gPC expansion was *a-posteriori* assessed by checking that the contribution of higher order polynomials remained very low for all considered quantities.

### Data Analysis

The effect of the outflow boundary conditions on computed hemodynamics was quantified by calculating the error between the simulation results and *in-vivo* data for flow rate and pressure drop ($\Delta P$) in $M_{\text{pre}}$ and $M_{\text{post}}$, both coupled with the 0D network, considering $\alpha = 0$, and the four different $\alpha$ values. The pressure drop is calculated as follows:

14 $$\begin{aligned} \Delta P(t) = P(t,\Gamma _{\text{in}}) -\; P(t,\Gamma _{\text{CoA}}) \end{aligned}$$

where $\Gamma _{\text{in}}$ corresponds to $\Gamma _{\text{pre}}$ and $\Gamma _{\text{post}}$ in case of $M_{\text{pre}}$ and $M_{\text{post}}$, respectively.

Since hemodynamic forces, particularly the wall shear stress (WSS), play an important role in the development and progression of vessel wall pathology, the effect of the $\alpha$ values was also evaluated in terms of Time Averaged Wall Shear Stress (TAWSS):

15 $$\begin{aligned} TAWSS = \frac{1}{T}\int _{0}^{T}\left| \mathbf{WSS} (s,t) \right| \cdot dt \end{aligned}$$

where *T* is the overall interval of the cardiac cycle, and *s* is the position on the vessel wall. TAWSS maps were computed for the whole cardiac cycle.

## Results

**Figure 2:**
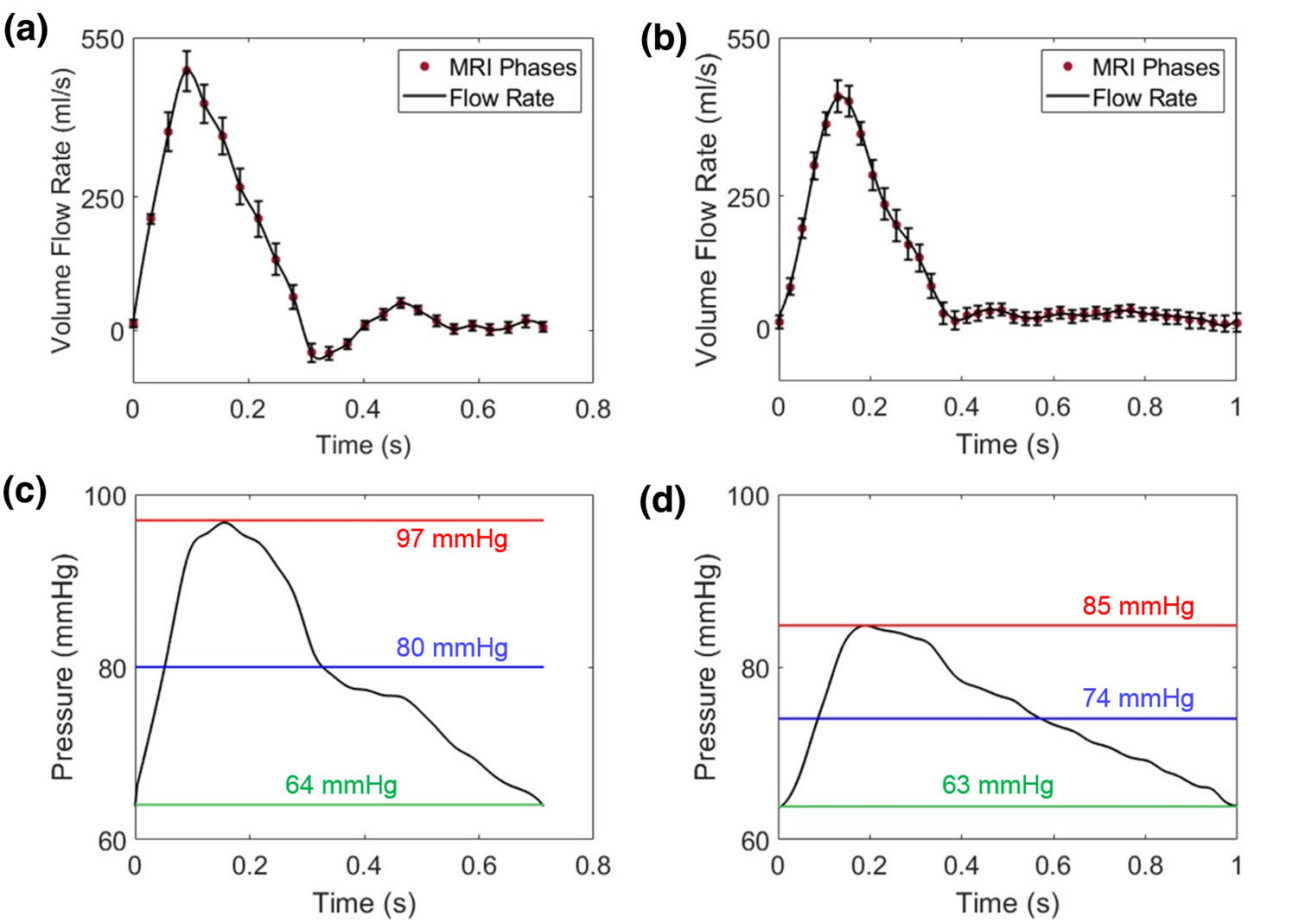
Flow rate waveforms extracted from the PC-MRI sequences at $\Gamma _{\text{pre}}$ (**a**) and $\Gamma _{\text{post}}$ (**b**) and related error bars due to the MRI inherent uncertainties; pressure curves at $\Gamma _{\text{pre}}$ (**c**) and $\Gamma _{\text{post}}$ (**d**) returned by the optimization algorithm. The lines overlapping the pressure curves indicate patient’s pressure values at $\Gamma _{\text{pre}}$ and $\Gamma _{\text{post}}$ reported in Table 1.

Figures 2a–2b show the mean time-dependent curve of the flow rate waveforms extracted from the PC-MRI sequences at the $\Gamma _{\text{pre}}$ and $\Gamma _{\text{post}}$ sections, respectively. The associated error bars of the intra-operator variability are also reported. The related pressure waveforms, at $\Gamma _{\text{pre}}$ and $\Gamma _{\text{post}}$ sections, obtained solving the optimization problem, are depicted in Figs. 2c–2d.

The computed pressure waveforms are consistent with the patient’s clinical conditions in terms of $P_{\text{max}}$, $P_{\text{min}}$ and $P_{\text{mean}}$. The values of $\overline{R}_p$, $\overline{R}_d$, and $\overline{C}$ for *pre-SP*, and *post-SP*, obtained by solving the optimization problem, are given in Table 2.

**Table 2:** $\overline{R}_p$, $\overline{R}_d$ and $\overline{C}$, for *pre-SP* and *post-SP *returned by the optimization algorithm. The resistances are expressed in $g\; cm^{-4}\; s^{-1}$, the compliance in $g^{-1}\; cm^{4}\; s^{2}$.

	$\overline{R}_p$	$\overline{R}_d$	$\overline{C}$
*pre-SP*	56.32	845.56	$1.06 \times {10^{-3}}$
*post-SP*	37.05	910.49	$2.3 \times {10^{-3}}$

The ${R}{_p}$, ${R}{_d}$ and the *C* computed according to 3WKM-total, and to 3WKM-tuning approaches for each outlets are reported in Table 3 and Table 4.

**Table 3:** 3WKM values for *pre-SP*. $R_p$ and $R_d$ are expressed in $g\; cm^{-4}\; s^{-1}$ and *C* in $g^{-1}\; cm^{4}\; s^{2}$.

		BCA			LCCA			LSA			DA		
		$R_p$	$R_d$	*C*	$R_p$	$R_d$	*C*	$R_p$	$R_d$	*C*	$R_p$	$R_d$	*C*
3WKM-total	$(\alpha =0)$	150.09	2253.87	6.00$\times 10^{-4}$	769.16	11550.06	1.17$\times 10^{-4}$	158.57	2381.19	5.68$\times 10^{-4}$	286.54	4302.77	3.14$\times 10^{-4}$
3WKM-tuning	$\alpha =-0.15$	127.58	1915.79	6.00$\times 10^{-4}$	653.79	9817.55	1.17$\times 10^{-4}$	134.79	2024.01	5.68$\times 10^{-4}$	1028.91	15450.58	3.14$\times 10^{-4}$
	$\alpha =-0.13$	130.58	1960.86	6.00$\times 10^{-4}$	669.17	10048.55	1.17$\times 10^{-4}$	137.96	2071.64	5.68$\times 10^{-4}$	736.48	11059.32	3.14$\times 10^{-4}$
	$\alpha =-0.10$	135.08	2028.48	6.00$\times 10^{-4}$	692.25	10395.05	1.17$\times 10^{-4}$	142.72	2143.07	5.68$\times 10^{-4}$	525.07	7884.67	3.14$\times 10^{-4}$
	$\alpha =-0.08$	138.07	2073.56	6.00$\times 10^{-4}$	707.63	10626.05	1.17$\times 10^{-4}$	145.89	2190.70	5.68$\times 10^{-4}$	444.61	6676.44	3.14$\times 10^{-4}$

**Table 4:** 3WKM values for *post-SP*. $R_p$ and $R_d$ are expressed in $g\; cm^{-4}\; s^{-1}$ and *C* in $g^{-1}\; cm^{4}\; s^{2}$.

		BCA			LCCA			LSA			DA		
		$R_p$	$R_d$	*C*	$R_p$	$R_d$	*C*	$R_p$	$R_d$	*C*	$R_p$	$R_d$	*C*
3WKM-total	($\alpha =0$)	194.87	4789.36	4.37$\times 10^{-4}$	342.23	8410.82	2.40 $\times 10^{-4}$	122.53	3011.36	6.95$\times 10^{-4}$	92.78	2280.27	9.18 $\times 10^{-4}$
3WKM-tuning	$\alpha =-0.15$	165.64	4070.96	4.37$\times 10^{-4}$	290.90	7149.21	2.40$\times 10^{-4}$	104.15	2559.66	6.95$\times 10^{-4}$	129.32	3104.47	9.18$\times 10^{-4}$
	$\alpha =-0.13$	169.54	4166.74	4.37$\times 10^{-4}$	297.74	7317.42	2.40$\times 10^{-4}$	106.60	2619.88	6.95$\times 10^{-4}$	119.69	2941.53	9.18$\times 10^{-4}$
	$\alpha =-0.10$	175.39	4310.42	4.37$\times 10^{-4}$	308.01	7569.74	2.40$\times 10^{-4}$	110.28	2710.22	6.95$\times 10^{-4}$	111.40	2737.94	9.18$\times 10^{-4}$
	$\alpha =-0.08$	179.27	4406.21	4.37$\times 10^{-4}$	314.85	7737.96	2.40$\times 10^{-4}$	112.73	2770.45	6.95$\times 10^{-4}$	106.75	2623.47	9.18$\times 10^{-4}$

### Effect of 3WKM Tuning on Flow Rate Waveforms

**Figure 3:**
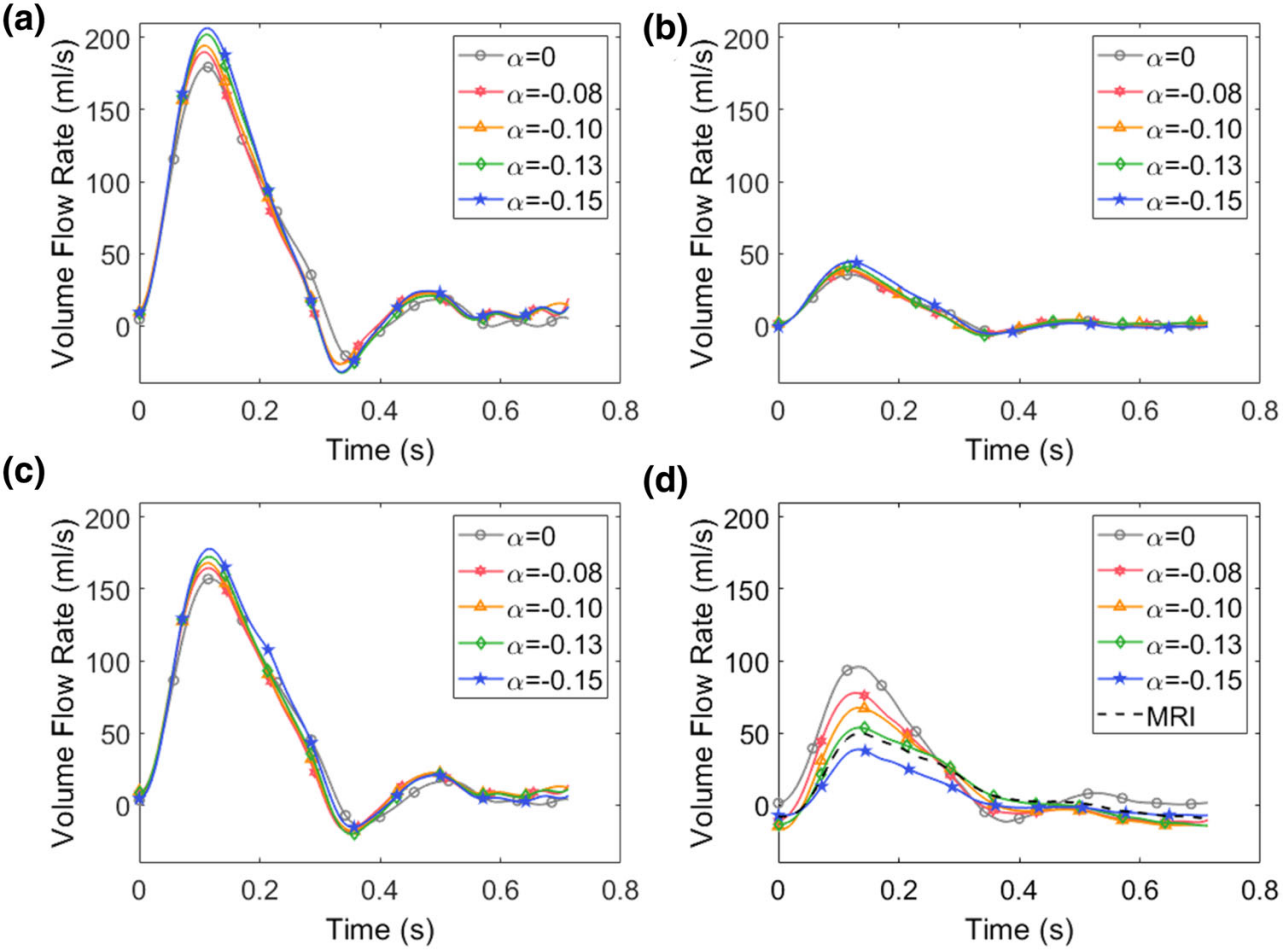
Flow rate waveforms corresponding to the *pre-SP* case. The effect of the different values of $\alpha$ is reported for BCA (**a**), LCCA (**b**), LSA (**c**) and $\Gamma _{\text{CoA}}$ (**d**) outlets. For $\Gamma _{\text{CoA}}$ section the PC-MRI flow is also reported (black dashed line).

**Figure 4:**
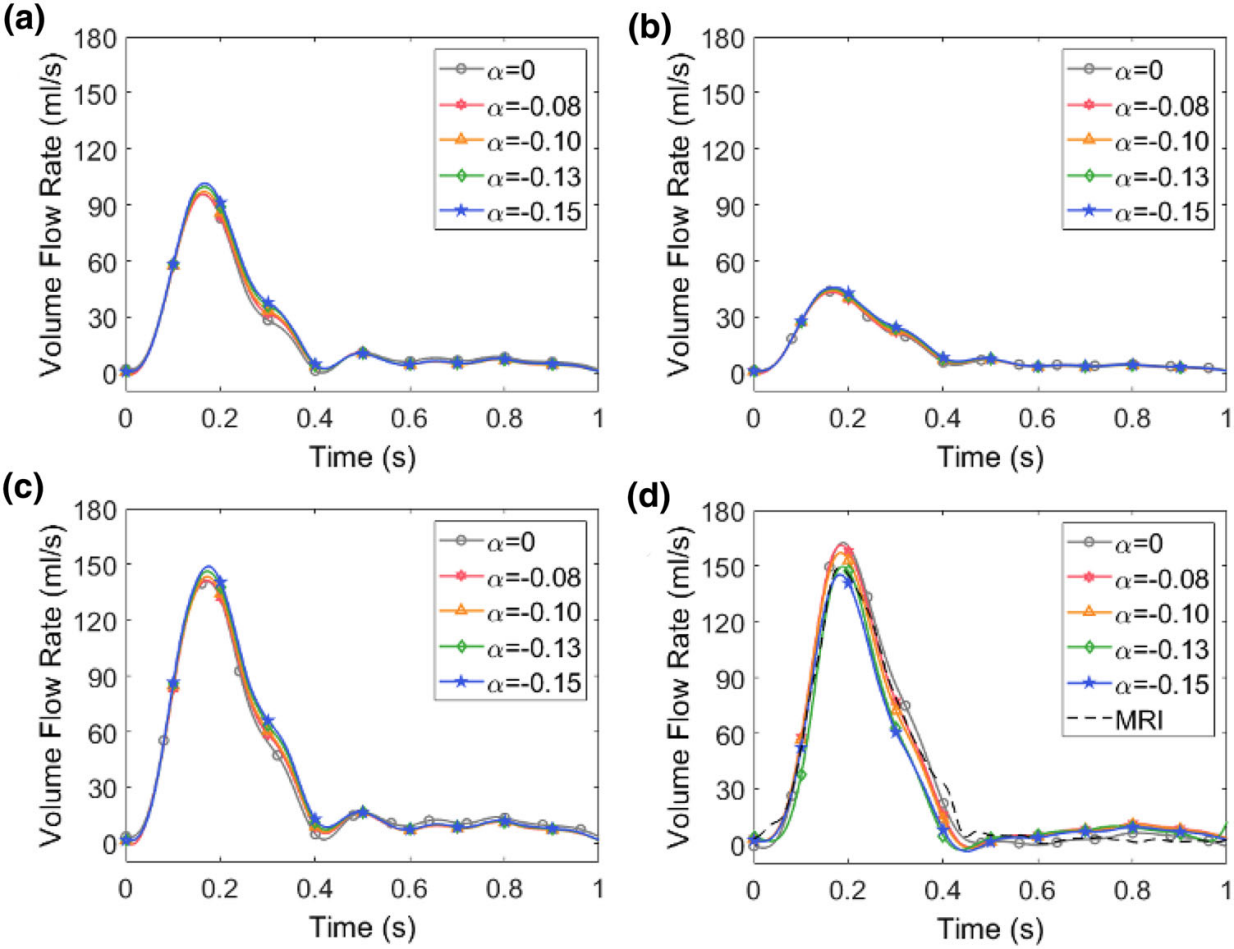
Flow rate waveforms corresponding to the *post-SP* case. The effect of the four different $\alpha$ is reported for the BCA (**a**), LCCA (**b**), LSA (**c**) and $\Gamma _{DA}$ (**d**) outlets. For $\Gamma _{DA}$ section the PC-MRI flow is also reported (black dashed line).

The flow rate partitioning at the aortic branches and control sections ($\Gamma _{\text{CoA}}$ and $\Gamma _{\text{DA}}$) are shown in Figs. 3 and 4 for *pre-SP* and *post-SP*, respectively. Both figures show the effect of $\alpha$: the flow rate waveforms for $\alpha = 0$ are superimposed on those obtained for the four deterministic simulations with the prescribed $\alpha$. Comparing the *pre-SP* and *post-SP* curves, we can observe a reduction in flow at the BCA and LSA outlets, and an increase in flow rate at $\Gamma _{\text{DA}}$, as consequence of the stenting procedure.

By visual comparison of the effect of $\alpha$ for all the simulated outflows, we observe that this parameter plays a more significant role in the *pre-SP* configuration, and that the maximum variability is found at the flow peak. This is particularly true at the level of coarctation/descending ($\Gamma _{\text{CoA}}$/$\Gamma _{\text{DA}}$). More in detail, Figs. 3d and 4d show the effect of $\alpha$ comparing the MRI to computational data: an error of approximately $96\%$ is found between the simulated blood flow obtained for $\alpha$ = 0 and that extracted from the PC-MRI at $\Gamma _{\text{CoA}}$. Indeed, when $\alpha$ = 0, the model overestimates the measured blood flow by 47 cm$^3$ s$^{-1}$. Analysing Fig. 4d, we always observe an overestimation of the flow for $\alpha = 0$, but slighter than in the *pre-SP* case. In particular, the model overestimates the measured flow rate by 13 cm$^3$ s$^{-1}$ when $\alpha =0$. The best agreement between simulated and measured flow rate waveform is achieved for $\alpha =-0.13$ (Fig. 3d) where we obtain a difference of 5 cm$^3$ $s^{-1}$ (equal to 6$\%$) between the two flow peaks (model and measured flow rates). This error percentage is reduced to 0.1$\%$ in the *post-SP* simulation.

The above results of deterministic simulations are complemented by the results obtained by applying the gPC method.

**Figure 5:**
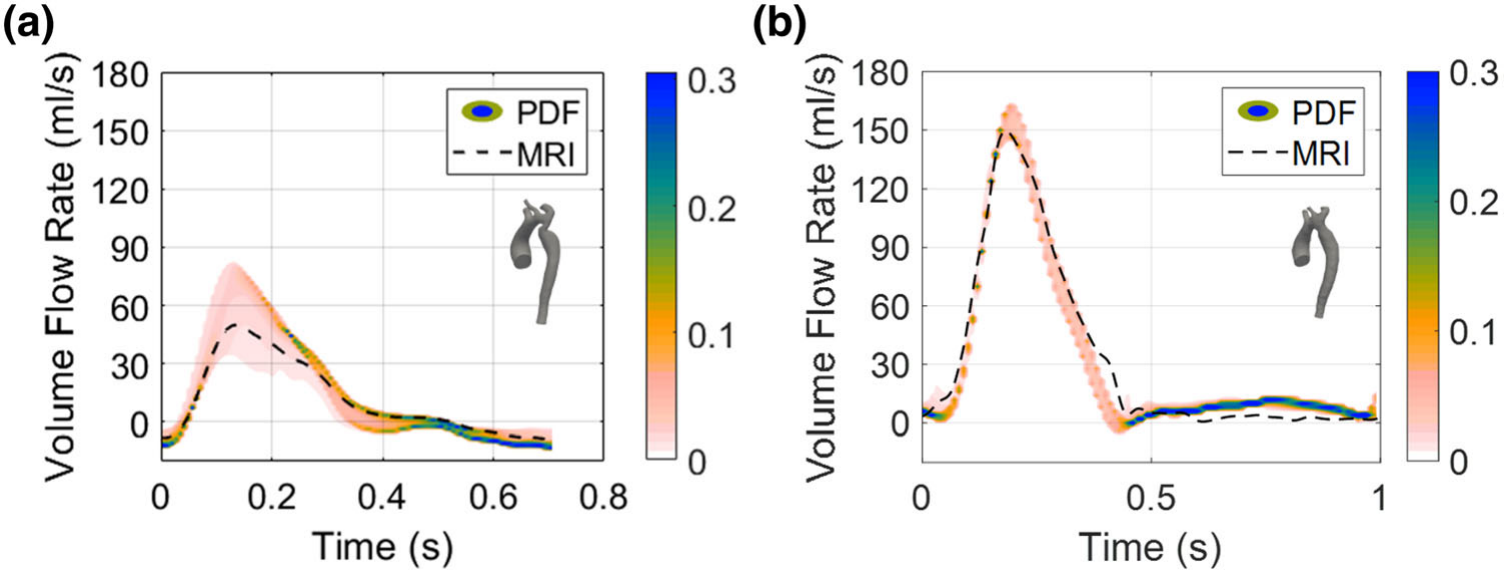
Stochastic probability density function of volume flow rate and MRI data (dashed line) at $\Gamma _{\text{CoA}}$ (**a**) and $\Gamma _{\text{DA}}$ (**b**).

In Fig. 5, the stochastic PDFs of the volume flow rate at $\Gamma _{\text{CoA}}$ (Fig. 5a) and $\Gamma _{\text{DA}}$ (Fig. 5b) are depicted with the *in-vivo* flow. The color bar represents the probability relative to the quantity of interest: the most likely part of the distribution is represented in blue, the least likely is represented in light pink.

The stochastic PDFs confirm that the greatest variability due to the effect of $\alpha$ is obtained in the *pre-SP* case, especially at the systolic peak. Inversely, the $\alpha$ parameter has a slighter effect on the flow rate waveforms of *post-SP* simulation.

### Effect of 3WKM Tuning on the Pressure Drops

**Figure 6:**
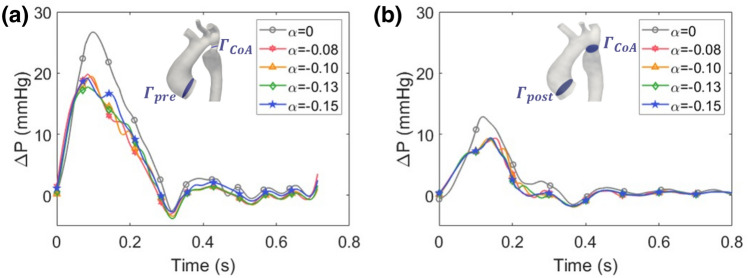
Pressure drop calculated for the $M_{\text{pre}}$ and $M_{\text{post}}$.

Figure 6 shows the computed aortic pressure drops for the two configurations $M_{\text{pre}}$ and $M_{\text{post}}$, together with the corresponding $\alpha$ values. In all cases, physiological pressure ranges are obtained. From a direct comparison between the two sets of graphs, we can observe a reduction of $\Delta P$ as the direct consequence of the stenting procedure. The maximum value of the pressure gradient is reduced from 20 to 10 mmHg, corresponding to the catheterization measurement reported in Table 1.

**Figure 7:**
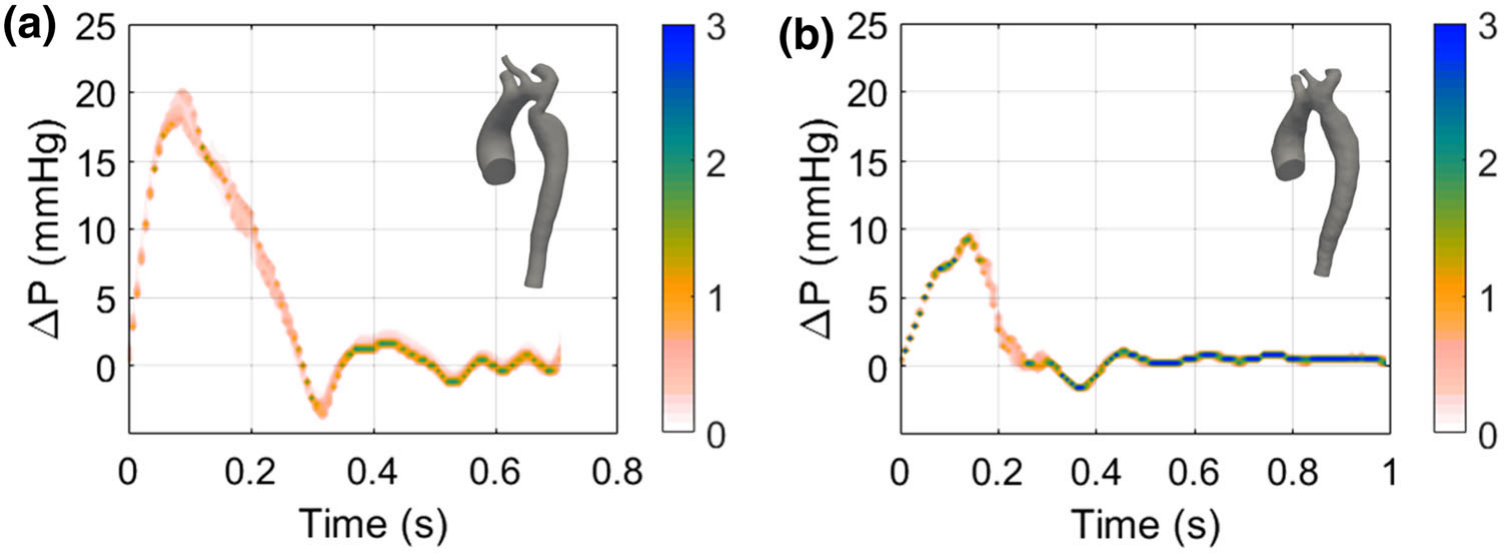
Stochastic probability density functions of pressure drop before (**a**) and after (**b**) stenting procedure.

At the systolic peak, the maximum pressure drop value obtained is 20 mmHg (corresponding to $\alpha =$ – 0.08), which differs by 2 mmHg from the value provided by the cardiac catheterization. In this case, the simulation underestimates the pressure. The pressure drop curves for $M_{\text{post}}$ show that there is almost complete overlap of the waveforms as the uncertain parameter varies, confirming a lower sensitivity of this model to the 3WKM tuning process (Fig. 6b). The finding that $\alpha$ does not significantly affect the pressure drop both before and after the stenting procedure is confirmed by Fig. 7, where stochastic PDFs of the pressure drop waveforms are shown. By analysing the effect of $\alpha$, we can observe that it is more significant for the flow waveform than for the pressure drop. This behaviour can be justified by the fact that the pressure drop is measured basing on the catheter position and not through the CoA. With this type of configuration, three outlets of the supra-aortic vessel are included in the measurement. Consequently, the measured pressure drop takes into account hemodynamic losses from all supra-aortic vessels in terms of pressure drop and WSS.

### Effect of 3WKM tuning on the stochastic TAWSS

**Figure 8:**
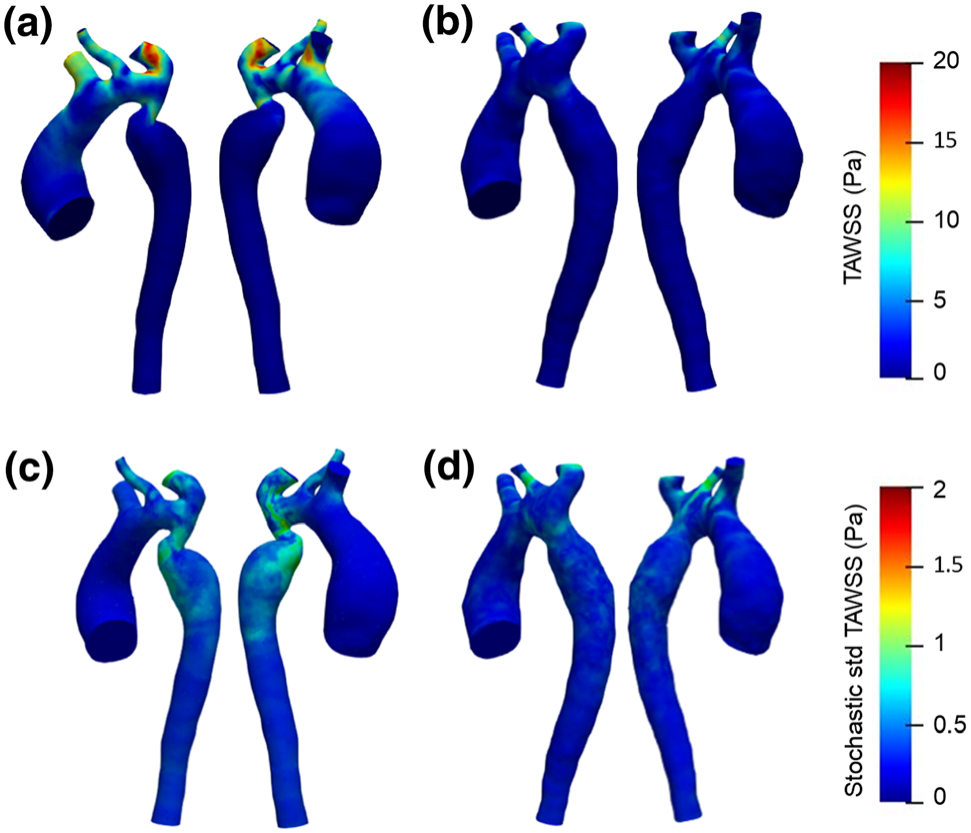
Nominal TAWSS maps calculated with $\alpha=-0.13$ for $M_{\text{pre}}$ (**a**) and for $M_{\text{post}}$ (**b**); stochastic standard deviation of TAWSS for $M_{\text{pre}}$ (**c**) and for $M_{\text{post}}$ (**d**).

Figure 8 shows the spatial distributions of the deterministic and stochastic standard deviation of TAWSS for both $M_{\text{pre}}$ and $M_{\text{post}}$ during the entire cardiac cycle.

The analysis of the deterministic case is reported for $\alpha$ = – 0.13. Figure 8a shows higher TAWSS values localised in the aortic arch, at the level of the supra-aortic branches, due to local curvature effects. In addition, high TAWSS values are present in the CoA region. Globally, a greater inhomogeneity in the TAWSS field is observed in the CoA region of the $M_{\text{pre}}$ (Fig. 8a) than in the $M_{\text{post}}$ (Fig. 8b), and regions near the CoA site have higher TAWSS values followed by lower TAWSS values downstream and on the outer surface of the vessel. The stochastic standard deviation distribution shown in Figs. 8c–8d are consistent with the PDF distribution reported in Fig. 5: increasing flow sensitivity to $\alpha$ leads to an increase in TAWSS standard deviation. It is worth noting that in *post-SP* there is a significant reduction in TAWSS as well as in its standard deviation (Figs. 8c–8d).

## Discussion

The choice of the boundary conditions is an important step in setting up a reliable CFD model of the cardiovascular system. The outlet boundary conditions, as well as the inlet boundary conditions, strongly influence the obtained flow patterns and hemodynamic parameters.^1^,^5^,^7^,^11^ PC-MRI sequences are widely used in the literature to retrieve suitable boundary conditions for CFD simulations and to validate computational models. Blood flow rate waveforms are frequently used to couple lumped 0D models to 3D models.^22^ In this context, the 3WKM is known to be an accurate representation of the downstream physical system, and most of the literature has focused on parameter estimation for the output boundary sections. However, defining the correct OBCs is not a trivial problem when considering complex physiological scenarios, such as in the case of severe coarctations.^11^,^14^,^16^,^19^,^21^ This work reinforces the need to refine outlet boundary conditions when modelling CoA cases. In addition, future works will explore the possibility of including them without the need of invasive measurement data. In this study, we evaluated the effect of 3WKMs on the flow rate, pressure, and TAWSS quantities. We processed PC-MRI acquisitions to extract time-varying flow rate waveforms that were imposed at the model inlet in both *pre-* and *post-SP* simulations. The flow rate waveforms extracted from MRI acquisitions at CoA plane were used to validate the simulation results. To limit uncertainties in the inlet boundary conditions, patient-specific time-varying velocity profiles were imposed at the model inlet in both *pre*- and *post-SP* simulations. However, imposing MRI-based velocity profiles can be also seen as another source of uncertainty due to MRI resolution that is currently unavoidable.

As pointed out in Sect. 4.1, an optimization approach gives more accurate results than fixing $R_p$, *C* and $R_d$ separately. As expected, the results show that the use of patient-specific pressure data, in combination with the optimization strategy, works more appropriately than evaluating the values to prescribe as boundary conditions basing solely on area measurements. By analysing Fig. 3, we can observe that in the case of $\alpha$ = 0, the flow error at the systolic peak is about 96$\%$. The error decreases to 8.7$\%$ in the post-intervention case (Fig. 4d). In the post-intervention case, the narrowed section has been restored, and the geometry is now comparable to a healthy case. In this configuration, our results are consistent with the overestimation of the systolic peak as reported by Pirola *et al*. 22. Comparisons of flow rates and PDF distributions with *in-vivo* data (Fig. 3d and Fig. 4d) showed that Windkessel-based method was able to ensure the expected flow. Considering $\alpha$ = – 0.13, a good agreement was reached between the simulated mean flow and that measured by PC-MRI at the $\Gamma _{\text{CoA}}$ and $\Gamma _{\text{DA}}$ sections in both $M_{\text{pre}}$ and $M_{\text{pre}}$. A reliable flow rate allows more accurate flow-related quantities to be estimated, such as TAWSS. However, accurate values of flow rate and flow-associated quantities are, in principle, hampered by the sensitivity of the several factors/assumptions. On the one hand, geometric reconstruction, in terms of local and global curvature, as well as the cross-section diameter, induces changes in the TAWSS. On the other hand, inaccurate flow reproduction introduces uncertainties in hemodynamic indicators.

Regarding the PDF distribution associated with these flow rate waveforms, it is worth noting that, at the systolic peak, there is the greatest variability in the output. This means that, at the systolic peak, the uncertainty and the variability of $\alpha$ have a large impact on the flow rate waveform. In diastole, in contrast, there is less variability, so the result is independent of the choice of $\alpha$. The graphs of the flow rate at $\Gamma _{\text{CoA}}$ (Fig. 3d) show a good agreement between the numerical results and *in-vivo* data. As for the *post-SP* case (Fig. 5b), $\alpha$ slightly influences the flow rate waveform in the overall cardiac cycle, confirming what was assessed by deterministic simulations. In contrast to the previous case, the greatest effect of $\alpha$ is in the diastolic phase. This result demonstrates that the tuning of 3WKMs based on $\alpha$ is not strictly necessary when dealing with physiological models of the aorta, as $M_{\text{post}}$. In this case, using patient-specific data as inlet boundary conditions and distributing the flows at the outlets, basing on the area ratio, as found in the literature, are an acceptable compromise to obtain reliable results of CFD simulations. It is also worth mentioning that in this study we adopted a stochastic analysis based on the gPC expansion theory with the advantage that computational costs are not dramatic as in case of previous studies were a larger number of simulations were used.^19^ Consequently, quantification of uncertainty may be useful to better understand the reliability of biomarkers obtained through numerical simulations, such as the pressure drop across the CoA site or TAWSS.

In this study, our focus was on the effect of the additional internal resistance added by the coarctation. In severe CoA, cross-sectional area estimation is particularly critical because of the low spatial resolution of the MR. This difficulty introduces a potential additional uncertainty in terms of geometry reconstruction similarly to those found in CT-based fractional flow reserve studies.^24^ In our formulation, since the adoption of the $\alpha$ parameter acts directly on the flow passing through the CoA cross-section, the geometric uncertainties are inherently included. The CFD models employed in this study involve several assumptions that should be noted. The main limitation may be that the analysis was performed considering only the images of a single patient. Although a single subject is insufficient to establish a new procedure, our methodology shows the possibility of obtaining time-varying outflow boundary conditions with additional constraints to account for CoA pressure drop. We described the aorta hemodynamics with rigid-wall models. Indeed, the patient-specific accuracy of FSI simulations requires reliable estimation of the structural constitutive parameters (e.g., vessel wall stiffness), which is nowadays rather difficult to achieve in clinics or the adoption of RBF-based strategies.^9^ Another possible limitation of the study could be that no additional control planes were acquired at the supra-aortic branches. Our approach did not impose any constraints at the level of BCA, LCCA, and LSA. This issue could be addressed by the adoption of 4D PC-MRI data. Moreover, this type of acquisition allows the investigation of potential local flow turbulence in a more accurate way.^2^ Pressure data obtained by cardiac catheterization were used, and it is well known that this is an invasive and risky procedure. Therefore, the use of pressure measured by alternative techniques is preferable in order to obtain a full non-invasive methodology.^3^

Our stochastic analysis was performed considering only one uncertain parameter, $\alpha$, which acts on the resistance of the systems. A previous study has shown that the capacitance *C* can also affect hemodynamic quantities of interest, such as flows, pressures, and WSS.^7^ The impact of *C* will be deeply investigated as future work, combining its effect with deformable-wall simulations. Finally, although the Newtonian rheological blood model is a well-accepted assumption, further investigation could be devoted to study the effect of non-Newtonian behaviour when severe CoAs are present.

## Conclusions

We showed that using the presented methodology, we reduced the flow error between *in-vivo* and *in-silico* data by fine-tuning the Winkdkessel model resistances at each outlet.The effect of the fine-tuning was pronounced in the case of CoA because of the additional resistance introduced by the coarctation especially at the systolic peak. In turn, $\alpha$ produced quite significant effects in the stochastic standard deviations of the TAWSS, evaluated on the systolic phase. The region of interest for TAWSS was different in the two cases: the area surrounding the CoA had the maximum of the variability simulating the *pre-SP* conditions; a region of LCCA had the maximum of the variability simulating the *post-SP* conditions. These results can contribute to the use of CFD for diagnostic purposes to obtain quantitative information, with known uncertainties, regarding pressure drop across the CoA site, thereby potentially reducing the need of invasive measurements in clinics.
